# Shoshin Beriberi Induced by Long-Term Administration of Diuretics: A Case Report

**DOI:** 10.1155/2014/878915

**Published:** 2014-07-03

**Authors:** Naoki Misumida, Hisashi Umeda, Mitsunori Iwase

**Affiliations:** ^1^Department of Cardiology, Toyota Memorial Hospital, 1-1 Heiwa-cho, Toyota 471-8513, Japan; ^2^Department of Medicine, Mount Sinai Beth Israel, First Avenue at 16th Street, New York, NY 10003, USA

## Abstract

Previous studies have suggested that diuretic therapy for heart failure may lead to thiamine deficiency due to the increased urinary thiamine excretion. Herein, we present the case of a 61-year-old man with shoshin beriberi, a fulminant form of wet beriberi, induced by long-term diuretic therapy. The patient had a history of heart failure with preserved ejection fraction and was receiving furosemide and trichlormethiazide therapy. He presented with worsening exertional dyspnea and was admitted for heart failure exacerbation. His condition failed to improve even after intensive treatment. A hemodynamic evaluation with the Swan-Ganz catheter revealed high-output heart failure with low peripheral vascular resistance. Thiamine was administered for suspected shoshin beriberi; his hemodynamic status improved dramatically within the next six hours. The serum thiamine level was below the normal range; the patient was therefore diagnosed with shoshin beriberi. The common causes of thiamine deficiency were not identified. Long-term diuretic therapy with furosemide and thiazide was thought to have played a major role in the development of thiamine deficiency. This case illustrates the importance of considering wet beriberi as a possible cause of heart failure exacerbation in patients taking diuretics, even when the common thiamine deficiency causes are not identified with history-taking.

## 1. Introduction

Beta-blockers and angiotensin converting enzyme inhibitors are now routinely used in the treatment of patients with chronic heart failure. Furthermore, nonpharmacological treatment has achieved marked progress. However, use of diuretics remains an important component of the treatment for moderate to severe chronic heart failure. Previous studies have suggested that diuretic therapy for heart failure may lead to thiamine deficiency due to the increased urinary thiamine excretion [1, 2]. Therefore, thiamine deficiency induced by furosemide is of particular interest beyond the spectrum of its well-known side effects. Thiamine is an essential cofactor in energy metabolism, and its deficiency may induce cardiovascular damage resulting in wet beriberi (beriberi heart disease), which is characterized by high-output heart failure with low peripheral vascular resistance. Herein, we present a case of shoshin beriberi, a fulminant form of wet beriberi, induced by long-term administration of the diuretics, furosemide, and thiazide.

## 2. Case Presentation

A 61-year-old man visited his primary care physician six months prior to admission with a complaint of exertional dyspnea for one month. Chest X-ray showed cardiomegaly and pulmonary vascular congestion. Echocardiography revealed a preserved ejection fraction with no significant valve disease. He was diagnosed as having congestive heart failure with preserved ejection fraction, and treatment was initiated six months prior to admission with medications including furosemide 40 mg/day, trichlormethiazide 1 mg/day, methyldigoxin 0.1 mg/day, and losartan 50 mg/day. His symptoms gradually resolved over the next several weeks. He developed slight myalgia in both thighs two months after the initiation of diuretics. Two weeks prior to admission, his exertional dyspnea recurred with gradual exacerbation; the patient therefore presented to our emergency department. His past medical history included diabetes mellitus and stage 3 chronic kidney disease secondary to diabetic nephropathy. He had no liver disease. The patient reported that he used to drink beer once a month and last consumed alcohol six months prior to admission. He also reported that he had consumed polished rice but had also been eating fruit and vegetables on a daily basis. The patient's family members endorsed his abstinence from alcohol and his daily fruit and vegetable consumption. The patient was not taking any vitamin supplements.

**Figure 1:**
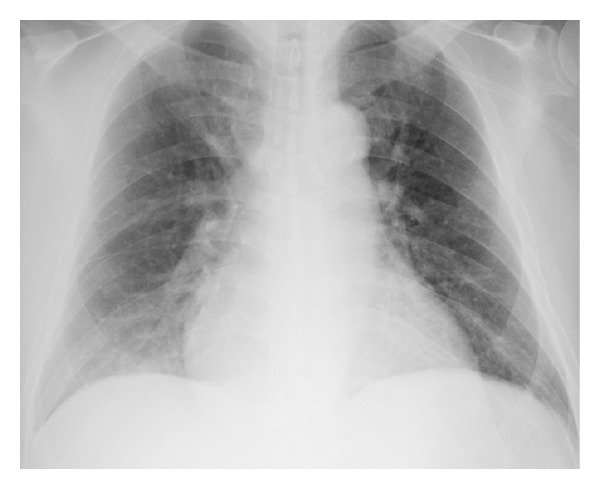
Chest X-ray shows cardiomegaly and pulmonary vascular congestion.

**Figure 2:**
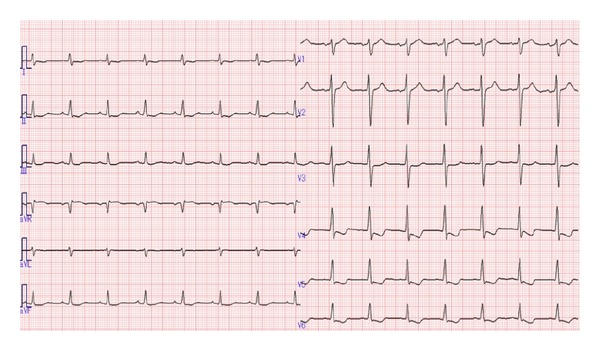
ECG shows downsloping ST depressions in left precordial and inferior leads.

**Figure 3:**
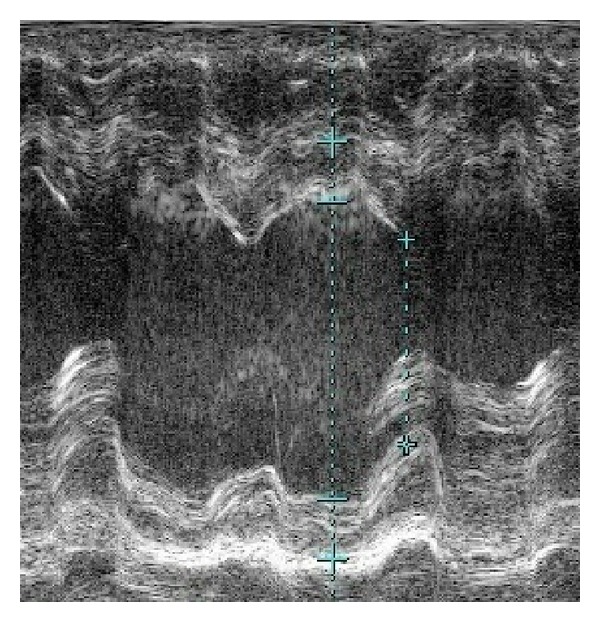
Echocardiography shows preserved left ventricular ejection fraction of 58% without regional wall motion abnormalities.

At the time of admission, he was in acute distress and was orthopneic. He was well nourished with BMI of 29. His blood pressure was 103/52 mmHg, which was lower than his usual approximate blood pressure of 140/90 mmHg. He had a heart rate of 92 bpm, respiratory rate of 30/min, and oxygen saturation of 98% on room air. Physical examination revealed jugular venous distention, but the heart sounds and breath sounds were unremarkable. Bilateral pitting edema was present throughout the legs, and prominent muscle tenderness was noted in both thighs. Chest X-ray revealed cardiomegaly and pulmonary vascular congestion (Figure 1). Electrocardiogram revealed downsloping ST depressions in left precordial and inferior leads (Figure 2). Echocardiography revealed a dilated left ventricle and preserved left ventricular ejection fraction of 58% without regional wall motion abnormalities (Figure 3). No other valvular lesions were found except for mild tricuspid regurgitation. Inferior vena cava was markedly dilated to 26.7 mm and the respiratory variation was diminished. Laboratory data are shown in Table 1. The atrial blood gas analysis revealed severe metabolic acidosis with a decreased HCO_3_^−^ level of 7.7 mEq/L and an elevated lactate level of 8.6 mmol/L; pH was maintained by compensatory hyperventilation suggested by the pCO_2_ level of 12.8 mmHg. Acute kidney injury was evident by the blood urea nitrogen and creatinine levels, 65 mg/dL and 3.7 mg/dL, which were remarkably elevated compared to his baseline levels of 20 mg/dL and 1.3 mg/dL, respectively. The creatine kinase level was also elevated up to 1,643 IU/L. Serum troponin I level was slightly elevated to 0.25 ng/dL, and the brain natriuretic peptide level was remarkably elevated up to 1,030 pg/mL.

The patient was admitted for heart failure exacerbation. Intravenous furosemide was administered repeatedly, but his urine output remained less than 10 mL/hour. His systolic blood pressure ranged from 80 mmHg to 100 mmHg, and a continuous infusion of dopamine was initiated. The metabolic acidosis persisted despite frequent administration of sodium bicarbonate. His condition failed to improve in spite of the abovementioned treatment. On the second day of admission, his systolic blood pressure dropped to 70 mmHg, and shortness of breath was exacerbated. He was transferred to the intensive care unit. The hemodynamic evaluation using the Swan-Ganz catheter revealed a cardiac index of 8.0 L/min/m^2^, pulmonary capillary wedge pressure of 27 mmHg, systemic vascular resistance of 266 dyne-sec*·*cm^−5^ (normal range: 700–1600 dyne-sec*·*cm^−5^), and mixed venous oxygen saturation of 78%. These findings were consistent with high-output heart failure with low peripheral vascular resistance. Given this hemodynamic profile and the existence of lactic acidosis, shoshin beriberi was strongly suspected, and thiamine (100 mg) was administered intravenously. His hemodynamic status improved dramatically within the next six hours with normalized diuresis. His blood urea nitrogen and creatinine levels returned to baseline, and the creatine kinase level was normalized within the following 48 hours. The hemodynamic profile 60 hours after thiamine administration was as follows: cardiac index, 4.3 L/min/m^2^; pulmonary capillary wedge pressure, 18 mmHg; systemic vascular resistance, 728 dyne-sec*·*cm^−5^ (normal range: 700–1600 dyne-sec*·*cm^−5^); and mixed venous oxygen saturation, 64%. Septic shock was excluded based on his overall clinical picture, absence of fever and leukocytosis, and negative results of serial blood cultures. Thyroid function was normal. The diagnosis of shoshin beriberi was confirmed by a low plasma thiamine concentration of 11 mg/dL (normal range: 20–50 mg/dL). Repeated careful neurological examination revealed horizontal nystagmus and hyporeflexia of bilateral lower extremities, and the patient was diagnosed with Wernicke's encephalopathy. The common causes of thiamine deficiency were not identified even with thorough history-taking. Intestinal malabsorption syndrome was excluded after gastrointestinal investigations including esophagogastroduodenoscopy and colonoscopy. His myalgia and nystagmus resolved within the next ten days, and he was discharged on day 15 with oral vitamin pills.

## 3. Discussion

To the best of our knowledge, this is the first case report of shoshin beriberi induced by long-term administration of diuretics. Although two cases of wet beriberi precipitated by diuretics have been reported previously, in both of these cases, the patient had a history of either gastrectomy or pancreaticoduodenectomy [3, 4]. In the present case, the common well-known causes of thiamine deficiency were not identified even with thorough history-taking and further investigations, and chronic diuretic therapy was thought to have played a major role in the development of thiamine deficiency.

Previous studies have suggested that diuretic therapy for heart failure may lead to thiamine deficiency due to the increased urinary thiamine excretion [1, 2]. The prevalence of thiamine deficiency in heart failure patients has been reported to range from 3% to 91% [5–10]. This wide variation is thought to stem from the differences in patients' background including their age, underlying diseases, and the assay validity of thiamine pyrophosphate activity. The prevalence is higher in patients of advanced age and with multiple comorbidities. A study that focused on outpatients with heart failure reported a very low prevalence of thiamine deficiency of 3% [8]. Interestingly, the largest cross-sectional observational study on 100 patients with heart failure found no relationship between thiamine deficiency and the furosemide dose [10].

Considering this high prevalence of thiamine deficiency in patients taking diuretics, it may seem odd that wet beriberi is not commonly seen in our daily practice and that there has been no reported case of shoshin beriberi induced solely by diuretics. This discrepancy may result from underrecognition of wet beriberi induced by diuretics due to the limited awareness of diuretic induced thiamine deficiency, and those cases might have been undiagnosed or diagnosed as shoshin beriberi of unknown etiology.

Urinary thiamine loss due to diuretic therapy is not specific to loop diuretics, and it has been shown to occur with virtually all diuretics in an experimental rat model [11]. Two different types of diuretics, furosemide and thiazide, were used concomitantly in this case, and this combination usage might have predisposed the patient to an even higher risk of thiamine deficiency compared to that with the usual furosemide monotherapy. In addition, the patient had stage 3 chronic kidney disease. A study that evaluated thiamine status in patients with stage 4 and stage 5 chronic kidney disease revealed that a substantial proportion of these patients had a high erythrocyte transketolase activity indicating a thiamine deficient state [12]. In the present case, the patient's underlying chronic kidney disease was not severe compared to those evaluated in the aforementioned study, but it might have contributed to the development of thiamine deficiency. However, the definite mechanism that led to shoshin beriberi in this patient, beyond the subclinical thiamine deficient state, remains unclear.

The present case had another interesting clinical characteristic of thiamine deficiency. The patient developed myalgia two months after the initiation of diuretics, which preceded the onset of worsening exertional dyspnea. The elevation of the creatine kinase level found upon admission was thought to be derived from the skeletal muscle rather than the heart because the elevation of troponin I level was subtle compared with the extent of the creatine kinase elevation. Myalgia has been observed in patients with beriberi neuropathy [13]. Myopathy with creatine kinase elevation has also been reported to be accompanied by thiamine deficiency, in which case myalgia preceded the development of heart failure [14]. Because of the temporal association of the symptom onset and diuretic administration, as well as complete resolution of the myalgia after thiamine administration, a final diagnosis of myopathy secondary to thiamine deficiency was made.

In the light of the high prevalence of thiamine deficiency in patients taking diuretics and its potential adverse effect on the cardiovascular system, the question is whether thiamine administration offers any benefit for these patients. Several studies have examined the association between thiamine supplementation and cardiac function measured by left ventricular ejection fraction in patients with heart failure. A pilot study conducted by Seligmann and colleagues evaluated the effect of intravenous thiamine on left ventricular ejection fraction in six patients with heart failure. It showed an improvement of left ventricular ejection fraction by 13% (from 24% ± 4.3% to 37.0% ± 2.4%) [5]. The first randomized, double-blind, placebo-controlled study [15], in which 30 patients were randomly assigned to one-week therapy with either IV thiamine or placebo, showed significant improvement of left ventricular ejection fraction at one week (from 28% ± 11% to 32% ± 9%, *P* < 0.05). After discharge, both groups of patients received oral thiamine for six weeks. At the end of seven weeks of the study, the left ventricular ejection fraction increased by 22% compared to baseline (*P* < 0.01). Recently, another randomized, double-blind, placebo-controlled study also reported a significant improvement of left ventricular ejection fraction after 28 days of oral thiamine administration [16]. These studies showed a promising effect of thiamine on left ventricular function in heart failure patients taking diuretics. However, this issue remains controversial because of their small study sizes and lack of a long-term followup.

While awaiting further studies and given the associated absence of toxicity and low cost, it would be reasonable to recommend a daily thiamine containing multivitamin to heart failure patients with a low thiamine level. Most importantly, heart failure caused by thiamine deficiency is treatable with thiamine administration, and a remarkable improvement can be expected as early as within 12 hours.

In conclusion, we report a case of shoshin beriberi induced by long-term administration of diuretics. This case illustrates the importance of considering wet beriberi as a possible cause of heart failure exacerbation in patients taking diuretics, even when the common causes of thiamine deficiency are not identified with history-taking.

## Figures and Tables

**Table 1:** 

WBC	11,000	/*μ*L
RBC	395	10^4^/*μ*L
MCV	100	fL
MCHC	34	%
Hb	11.1	g/dL
Plt	23	×10^4^/*μ*L
BUN	65	mg/dL
Cre	3.7	mg/dL
Na	135	mEq/L
K	5.0	mEq/L
Cl	95	mEq/L
Ca	8.8	mEq/L
CK	1643	IU/L
Troponin I	0.25	IU/L
AST	35	IU/L
ALT	35	IU/L
T-bil	0.6	mg/dL
LDH	327	IU/L
ALP	114	IU/L
TP	6.5	g/dL
Alb	3.9	g/dL
CRP	0.8	mg/dL
Glu	217	mg/dL
HbA1c	6.4	%
BNP	1030	pg/mL
pH	7.399	
pCO_2_	12.8	mmHg
pO_2_	99.3	mmHg
HCO_3_^−^	7.7	mmol/L
Base excess	−14.4	mmol/L
Anion gap	30.3	mEq/L
Lactate	8.6	mmol/L
